# Spiritual comfort, spiritual support, and spiritual care: A simultaneous concept analysis

**DOI:** 10.1111/nuf.12845

**Published:** 2022-11-30

**Authors:** Ana Patrícia Tavares, Helga Martins, Sara Pinto, Sílvia Caldeira, Patrícia Pontífice Sousa, Beth Rodgers

**Affiliations:** ^1^ Universidade Católica Portuguesa, Institute of Health Sciences Palma de Cima Lisbon Portugal; ^2^ Universidade Católica Portuguesa, Institute of Health Sciences, Centre for Interdisciplinary Research in Health Palma de Cima Lisbon Portugal; ^3^ Instituto Politécnico de Beja Beja Portugal; ^4^ CINTESIS@RISE, Nursing School of Porto (ESEP) Porto Portugal; ^5^ Chair Adult Health and Nursing Systems Department, School of Nursing Virginia Commonwealth University Richmond Virginia USA

**Keywords:** nursing, simultaneous concept analysis, spiritual care, spiritual comfort, spiritual support

## Abstract

**Background:**

Spirituality is a dimension of life and the human being that should be included in holistic healthcare. One major barrier often described by nurses on implementing spirituality in practice relates to perceiving the concept of spirituality as subjective and sharing confounding similarities with other concepts. In this sense, the concepts of spiritual comfort, spiritual care, and spiritual support may require more distinct theoretical definitions aimed at clear and effective nursing interventions within spiritual care.

**Aim:**

To provide a definition of spiritual comfort, spiritual support, and spiritual care.

**Methods:**

Simultaneous concept analysis (SCA) of three concepts according to Haase et al., which is grounded on Rodgers' evolutionary view. The method was based on a literature review with the search of electronic databases on May 2020. Search and analysis have been blinded conducted by two reviewers.

**Results:**

One hundred thirty‐six studies were included in the SCA. Findings suggest that spiritual comfort is an immediate state and an outcome. Spiritual support is related with an intimate and positive relationship with God. Spiritual care is defined as a complex and interactive process. Both spiritual support and spiritual care are grounded in a therapeutic context.

**Conclusion:**

This SCA allowed the attributes of each concept to be identified and provides definitions that may facilitate the understanding of these concepts and promote the implementation of spirituality in nursing practice, but which has also led to future research on this topic.

## 1 BACKGROUND

Spirituality is recognized as a fundamental dimension of life that can contribute positively to patients' health and well‐being and in dealing with illness and diseases.^1^ Understanding spirituality in nursing may start with the recognition of this dimension as part of the global human being in a context of holistic nursing care. Spirituality represents a broad concept, often described as a subjective and multidimensional component of being human.^2^

Spirituality is broader than religiosity, and has been defined in nursing as “(…) a way of being in the world in which a person feels a sense of connectedness to self, others, and/or a higher power or nature; a sense of meaning in life; and transcendence beyond self, everyday living, and suffering.”^3^ ^,p.93^ Despite being defined, the concept seems to be far from being integrated into practice and the barriers in the delivery of spiritual care by nurses are already described in the literature. The main barriers are education and training on spiritual care, lack of time, and clear guidelines regarding spiritual care, and the difficulty of defining spirituality and spiritual concepts because of their subjectivity.^4^ Due to the subjectivity of the concept often reported by nurses, discussing the boundaries between personal belief and professional practice is still a difficult task.^5^ The subjectivity of the concept and considering other concepts as similar may affect the implementation of spiritual care at the beginning of the process of being aware of the meaning of spirituality and related concepts. Concept development therefore seems to be essential in the process of clarifying concepts before being used in practice, but also for use in research.

In this regard, the concepts of spiritual comfort, spiritual support, and spiritual care are examples of the scope the subjectivity of spiritual dimension may have and of the gray areas among concepts, which may reflect on nursing care. Spiritual care and spiritual support have been studied more frequently than spiritual comfort, which remains poorly analyzed and defined.

Comfort is a subjective concept and has been analyzed from different perspectives. Kolcaba,^6^ in her theory about comfort, defines the concept as an immediate state of being strengthened by the feelings of relief, tranquility, and transcendence satisfied in four contexts (physical, psychospiritual, social, and environmental). Although the spiritual dimension of comfort is explicit in this theory, the concept remains mainly associated with promoting physical care, such as hygiene care (and comfort), pain relief or positioning, or by the absence of comfort care in other dimensions that are perceived by patients as critical, specifically in nursing practice.^7^, ^8^ The physical domain of comfort is related to the gaps and inconsistencies based on the difficulty of measuring the concept, and the association with physical emphasis of the concept persists mainly in daily care practice. Current scientific evidence also demonstrates this limitation. Further studies that develop the knowledge of comfort in other dimensions, such as the spiritual dimension, are needed.

Spiritual care as a concept is related to implementing spirituality and is grounded in the use of self of “being there” or of the presence of the healthcare provider which brings hope and peace to the patients and families.^9^ Spiritual support is considered a nursing intervention and is included in the Nursing Intervention Classification; it is defined as “assisting the patient to feel balance and connection with a greater power.”^10^ ^,p.1199^

These concepts share similar attributes not only in the spiritual dimension, but in the understanding of comforting, supporting and caring that are common to other areas. A conceptual clarification may facilitate the understanding and implementation in clinical practice, particularly in assessing and addressing patients' spiritual needs. There is missing in the literature a clear definition of spiritual comfort, to make a clear distinction between the concepts. This clarification that this paper intends to give may help both theory and practice.

The simultaneous analysis of concepts (SCA) procedure was developed by Haase et al.,^11^ and allows the clarification and development of related concepts in addition to the potential for strengthening the professional language. This method is based on Rodgers' evolutionary view of concept development.^12^ SCA “is designed to analyze interrelationships and identify theoretical overlap, common themes, and distinguishing characteristics among similar or complementary concepts.”^11^ ^,p.227^ Therefore, the aim of this study is to achieve a clear conceptual definition and a greater understanding of the individual meaning of spiritual care, spiritual support, and spiritual comfort as singular concepts, to provide exclusive theoretical definitions for each one, and to highlight their interrelationships and distinguish characteristics.

## 2 METHODS

This is a SCA study, composed of the nine steps proposed by Haase et al.,^11^ which is grounded on Rodgers' evolutionary view. SCA model is based on the rationale that many concepts cannot be individually analyzed due to interconnections among concepts. They can, however, be understood by related comparison, since concepts are often similar enough that those closely associated need to be addressed. This is the situation with the concepts of spiritual care, spiritual comfort, and spiritual support.

### 2.1 Data sources

Literature search was performed in May 2020, focused on spiritual care, spiritual comfort, and spiritual support on the following databases: Cumulative Index to Nursing and Allied Health Literature (CINAHL), Academic Search Complete, Medical Literature Analysis and Retrieval System Online (MEDLINE), Scientific Electronic Library Online (SciELO), and Scopus. CINAHL and MEDLINE were chosen to explore nursing, medicine, and health related literature. SciELO and Scopus are scientific databases that encompass literature that goes beyond health literature and may provide some contributions to this concept analysis from other areas. The terms used in the databases were “Spiritual Care,” “Spiritual Comfort,” and “Spiritual Support,” and were searched to identify literature with those terms in the title. The search was conducted in each database and no time filter was used. This decision is related to the need to establish a conceptual foundation for each concept as complete as possible, retrieving the most information possible. Details of the search strategy and search results are presented in Table 1.

**Table 1:** Number of citations on the databases search and included articles

	CINAHL	MEDLINE	Academic search complete	SciELO	Scopus	Total	Number of included articles
Spiritual care	1143	913	716	17	247	3036	103
Spiritual support	85	64	71	1	96	317	27
Spiritual comfort	8	3	11	1	11	34	4

*Note*: t available electronically

Research papers written in English, Spanish, or Portuguese that discussed spiritual care, spiritual comfort, or spiritual support directly, including those that provided oblique definitions, antecedents, or attributes of each concept were included in this review.

A total of 3036 articles was identified specifically about spiritual care, 317 about spiritual support, and 34 about spiritual comfort. After the blinded screening, 4 articles specifically on spiritual comfort, 28 articles on spiritual support, and 104 articles on spiritual care were included in the SCA. PRISMA flow for each individual concept is available in Supporting Information: Appendix A.

### 2.2 Data analysis

According to Haase et al.,^11^ the purpose of data analysis is not to develop a complete and finished definition of the concepts, but to provide an initial point for continued conceptual exploration in the discipline of nursing. Thematic analysis was performed as suggested by Haase et al.^11^ and each article was read as often as necessary to determine the attributes, antecedents, and outcomes from each concept, that precedes the construction of the validity matrix. These aspects of three concepts were identified based on a rigorous analysis and the process of information collection was systematic, confirmable, and repeatable. In fact, attributes, antecedents, and outcomes of the three concepts were extracted from relevant articles and compared between and across disciplines before creating a validity matrix for comparison. In the next step, critical attributes, theoretical definitions, antecedents, and outcomes were derived for each concept independently.

## 3 RESULTS

### 3.1 Spiritual comfort

Four papers were analyzed on spiritual comfort. Despite no clear definition of the concept being found in that literature, spiritual needs were reported to have implications for patients' comfort needs. Spiritual comfort is described as a result of the perception of the patient, which means that it relates to inner consciousness of self. It is an immediate state and an outcome that establishes spiritual comfort as a goal from nursing interventions. As presented in the literature it becomes clear that providing spiritual comfort requires effective communication. Regarding the attributes of the concept, we realized that spiritual comfort is related to connection with self, others, environment, superior entity, God, or the transcendent. When a patient experiences spiritual comfort, he/she obtains a sense of inner peace, well‐being, and feels supported by family and healthcare professionals. No enablers for the spiritual comfort concept were identified in literature. The process model of spiritual comfort is explained in Table 2.

**Table 2:** Process model: Spiritual comfort

Antecedents		Attributes	Outcomes
Awareness of religious and spiritual needs		Connection with self, others, environment, superior being, or God	Feeling supported by family and healthcare professionals
Effective communication		Gratitude to God	Sense of inner peace
Emotional support		Immediate state	Well‐being
Holistic care		Nurturing environment	
No unresolved (or unfinished) issues		Outcome	
Symptom management		Perception of the patient	
Patient‐centered care		Spiritual and religious practices	
Patient‐healthcare team relationship		Transcendence	
Prevention of social isolation			
Spiritual and religious beliefs			
Surrogate terms		Spiritual health Well‐being	
Obstacles		Anger Depression Existential issues Extreme pain Fear Guilt Hopelessness Shame Spiritual pain	
Definition		Spiritual comfort concept is a state and an outcome perceived by the patients and is grounded in holistic and patient‐centered care, a nurturing environment and in spiritual and religious beliefs, which have a positive impact on patients' well‐being and inner peace.	

### 3.2 Spiritual support

Spiritual support was described in the literature reviewed as being potentially therapeutic in nature. In fact, spiritual support was discussed as based on a holistic approach of patients' needs, which is grounded in an affective relationship and communication between patient and staff. To achieve an effective communication, it is mandatory to establish a trustful relationship and empathic caring.

One of the attributes of spiritual support is the perceived positive influence of the concept by patients, also spiritual support is an intrapersonal concept. However, when we deeply analyze the several attributes of spiritual support, we can perceive that the concept is very focused and related to the intimate and affectionate relationship with a higher power or God, and closeness to the sacred or transcendent.

The results on spiritual support are displayed in Table 3.

**Table 3:** Process model: Spiritual support

Antecedents	Attributes	Consequences
Awareness of needs of the patient	Advanced care planning	Comfort
Awareness of religious or spiritual resources	Closeness and connection (perceived support from God to the sacred or transcendent, family members and friends, and healthcare professionals)	Coping with illness Positive impact on the healing process Quality of life Resilience Satisfaction with care Sense of security Social cohesiveness Spiritual transformation Terminal illness awareness Well‐being
Effective relationship and communication between patient and healthcare team	Intimate, affectionate, and personal supportive relationship with a higher power or God	
Holistic approach	Intrapersonal phenomenon and perceived positive influence	Improve mental health
One's spiritual and religious beliefs and practice	Perceived social–environmental resource	Improving sense of self
Sensitive and knowledgeable in assessing an individual's religious, spiritual and cultural beliefs, and value systems	Therapeutic nature	Meaning and finding purpose
Surrogate terms	Existential support Religious support Social support Spiritual assistance Spiritual counseling Spiritual professionalism	
Obstacles	Concordance/discordance of spiritual beliefs Discomfort talking about spirituality Inability to express needs Inadequate training and skills/competence Insufficient resources Neglect spiritual Taboo subject	
Enablers	Adequate environment for spiritual support Be authentic Concordance/discordance of spiritual beliefs Time available	
Definition	Spiritual support concept is an intrapersonal phenomenon that results from the perception of support from God, so this therapeutic, intimate, and affectionate relationship with a higher power or God may have a positive impact on the patient in a sense of security, comfort, and resilience.	

### 3.3 Spiritual care

According to the literature, spiritual care represents a complex and multifaceted phenomenon with a therapeutic nature. The process of spiritual care delivery is interactive and intentional and begins with the identification and assessment of spiritual needs of the patients. The relationship between patient and nurse must be based on trust and a multidisciplinary team approach, as this is vital for promoting patients' feelings of inner peace, gratitude, hope, comfort, and spiritual well‐being. The literature emphasizes the role of nurses as key elements in the providing of spiritual care. The intuitive sense, the spiritual/transcendent, and self‐awareness must be developed as well as moral skills.

The results are summarized in Table 4.

**Table 4:** Process model: Spiritual care

Antecedents	Attributes	Outcomes
Identification and assessment of spiritual needs	Advanced nursing practice	Adherence of treatments
Moral skills	Complex	Comfort
Multidisciplinary team approach	Ethics	Cooperation
Perception and sensitivity of spiritual care	Fostering the search for meaning	Coping
Professional commitment	Harmonious connection with self, others, and/or God or superior being or transcendence	Dignity
Religious/spiritual rituals	Holistic, interactive, interpersonal, and intentional patient‐centered care	Healing
Respecting and supporting spiritual/religious beliefs Spiritual/transcendent self‐awareness (nurse and patient)	Intimate	Hope Improve mental health Nurses' satisfaction
Therapeutic communication	Intuitive sense	Pain management
Trust relationship between nurse patient	Mostly subjective	Quality of care
	Multifaceted phenomenon	Quality of life
	Therapeutic (environment or use of self)	Spiritual growth
		Spiritual health
		Spiritual integrity Spiritual well‐being/well‐being
Surrogate terms	Religious assistance spiritual assistance spiritual caring Spiritual healing Spiritual service Spiritual support	
Obstacles	Absence of guidelines Conflict with institutional practices Cultural discordance Difficulty in recognizing patients' spiritual care needs High workload Hospital environment and task‐oriented care Inadequacy of human resources Lack of motivation, time, knowledge/training, support, confidence, privacy, objectivity of the concept of spiritual care, personal religiousness, and focus on patients Neglect of spiritual dimension Not giving timely response Nurse discomfort Uncertainty about the role	
Enablers	Being confident in their spirituality Education and training in spiritual care Self‐satisfaction Supportive environment	
Definition	Spiritual care concept is an interpersonal and multifaceted phenomenon with a therapeutic nature based on a trust relationship between nurse and patient that requires intentionality, respect, and professional commitment aiming to promote hope, inner peace, quality of life, and comfort.	

### 3.4 Validity matrix for critical attributes

The construction of validity matrix^11^ intends to explore interrelationships between concepts to identify and display commonalities across concepts. In this step, it is necessary to consider each element considering others, through constant comparisons and contrasts to identify inconsistencies and gaps. As shown in the validity matrix, spiritual care, spiritual support, and spiritual comfort are concepts with similarities but which also have significant differences (Table 5).

**Table 5:** Validity matrix of critical attributes of spiritual care, spiritual comfort, and spiritual comfort

	Spiritual comfort	Spiritual support	Spiritual care
Action	Immediate state	Process and internal resource	Interactive and intentional process
Characteristics	Perception of the patient	Intimate Affectionable relationship with higher power or God	Altruistic Compassion Complex Intimate Mostly subjective Personal
Environment	Nurturing environment	Perceived social–environmental resource	Healing environment Nurturing environment Therapeutic environment
Ethical orientation	Respect and dignity	Dignity, respect, ethic	Dignified care; is an ethic
Extra personal issues	Connection with others, environment, or God Gratitude to God spiritual, and religious practices Transcendence	Closeness to the sacred or transcendent Interconnectedness with a greater connection with God, members of religious communities, family members and friends, and healthcare professionals Power/God	Connection with others, superior being and/or God
Intrapersonal issues	Connection with self	Connection/relationship with higher power or God	Connection with self
Nature	Outcome	Therapeutic nature Intrapersonal	Therapeutic nature Interpersonal and multifaceted phenomenon Holistic and patient‐centered care Mostly subjective Compassion and dignified care
Nurse skills			Just being there Intuitive sense Presence Therapeutic use of self
Nursing		Advanced care planning	Advanced nursing practice Nursing spiritual care intervention
Responses		Perceived positive influence for the patient	Healing

Spiritual comfort is connected to an outcome that may result from nursing interventions. Spiritual support and spiritual care are grounded on a therapeutic nature, however spiritual support is related to an intrapersonal and transcendental dimension while spiritual care is related to interpersonal nature. It means that spiritual support relates to an intimate and affectionate relationship with a higher power or God and spiritual care relates to connection with self and others.

The detailed results including the studies of this SCA can be found in Supporting Information: Appendix B.

## 4 DISCUSSION

This SCA was focused on three concepts related to spirituality that needed further analysis and clarification. This analysis is bringing the attributes, the antecedents, and the consequences of each concept and, in addition, disclosed the similarities and differences between the three concepts. Spiritual comfort, spiritual support, and spiritual care are interrelated concepts, and this is the main rationale of this review. Concept analysis is growing in some particular dimensions of nursing knowledge, and regardless of some criticism, some authors reinforce the need to conduct these studies to achieve an effective definition of a concept to promote nursing science and clinical care.^13^ It is clear that the difficulty in defining the proposed concepts is not a contemporary problem, that is, it lies above all in the definition of the attributes of the process of caring, supporting and comforting, which are not yet clear in the literature, therefore, this difficulty is a current reality in different contexts.

In these findings, spiritual comfort is defined as an immediate state and an outcome. However, this concept is still insufficiently developed for application and testing and only slightly operationally implemented in clinical practice, on this topic. Pinto et al.^14^ found it difficult to define and operationally implement comfort, despite it being a fundamental need of human life and a vital concept in nursing care and philosophy. These authors underline that many definitions of comfort do not include the spiritual dimension, and this can be a reductionist approach of the concept since comfort needs to be embraced in a holistic perspective.^14^ Considering this need, this study proposes a new definition of the concept that includes the spiritual dimension, which allows a broader concept perspective.

Spiritual support is more focused on an intimate and personal relationship with God and seems to be mainly related to the vertical dimension of spirituality. This connection with God or a superior being or entity is perceived as something positive. Several studies reinforce the positive relationship with God as a source of love, hope, coping with difficult times, and improves spiritual well‐being, much needed in times of illness and crisis.^15^, ^16^, ^17^

The results concerning the analysis of the concept spiritual care are similar to Ramezani et al.^18^ In what concerns the attributes of the concept of spiritual care, the intuitive sense, therapeutic nature, and the therapeutic use of self, have also been found to be critical attributes in this study. The therapeutic use of self is considered a core element in spiritual care and contributes to effective holistic care. The use of self is a step forward in the way of “*being*” in the provision of spiritual care by health professionals.^19^ The antecedents of sensitivity and awareness regarding spiritual care had already been referenced in previous analyses of the concept. It is essential for health professionals to be aware of their own spirituality to feel more comfortable talking to patients about this topic, which has a personal and intimate perspective.

The analysis of these three concepts found similarities and differences, that allows a differentiating framework to be established. The concept of spiritual comfort is reported as an immediate state and an outcome, that is a consequence of a nursing intervention. We found that both the spiritual care and spiritual support concepts have the comfort dimension as a consequence, which is congruent with previous findings. It means that spiritual care and spiritual support could be encompassed into the scope of interventions and spiritual comfort into the outcomes. When we look into the antecedents it becomes clear that the identification and assessment of the spiritual needs of the patients is essential in the three concepts.

Spiritual comfort, spiritual care, and spiritual support are vital in the spiritual approach of patients, nevertheless the spiritual dimension is still neglected in nursing care and must have the same focus and importance as the other human dimensions. It refers to the holistic dimension of nursing care that should embrace the whole person and include the spiritual dimension, as we can find in the antecedents of the three concepts.

Spiritual comfort is an immediate state and a result of the perception of the patient, that emerges from the connection with self, others, environment, and something superior or God. It is a positive feeling that brings a sense of inner peace, well‐being, and feelings of being supported. In the same way, both spiritual care and spiritual support are seen as bringing well‐being and feelings of inner peace, love, and quality and satisfaction with life.

The differences between the concepts of spiritual support and spiritual care, lies in the nature. Spiritual support has an intrapersonal nature, that refers to the intimate and affectionate relationship with a higher power or God, refers to an interconnectedness and a connection with God, others, and environment, but it also refers to an interpersonal phenomenon. It means that spiritual care is an interactive and intentional process that includes, beyond the intimate and affectional connection with self, others, or God, the nursing intervention that is holistic and patient‐centered.

The validity matrix of critical attributes of spiritual care, spiritual comfort, and spiritual support outline that the ethical orientation is present in all three. Caldeira and Timmins^20^ emphasize that the provision of spiritual care by nurses involves an ethical approach that is grounded on being respectful and truthful, maintaining confidentiality, and providing dignity while preserving care. In addition, all ethical principles must be present in spiritual care, but beneficence is considered the most important when delivering spiritual care.^21^

This review brings into discussion the need of further research on these concepts, such as studies comprising patients' experiences and perspectives.

## 5 CONCLUSION

This study enhances the conceptual understanding by clarifying the concepts of spiritual comfort, spiritual support, and spiritual care. Our finding shows that spiritual support and spiritual care are grounded in a therapeutic nature in which one of the outcomes is the immediate state of spiritual comfort. Spiritual support mainly encompasses the vertical dimension of the spiritual in which there is an intrapersonal and intimate relationship with God. The therapeutic nature of spiritual care is a unique, multifaceted, interpersonal, and complex process, besides being broader and more widespread than spiritual support because it embraces the vertical and horizontal dimension of spirituality. Although this inquiry contributed to definitions of the concepts, there is the need to foster the development of this field and research regarding this topic with a goal of enhancing nursing science and care.

## AUTHOR CONTRIBUTIONS

*Substantial contributions to conception, data collection, data analysis, and writing*: Ana Patrícia Tavares, Helga Martins, Sílvia Caldeira, and Beth Rodgers. *Drafting the article or revising it critically for important intellectual content*: Ana Patrícia Tavares, Helga Martins, Sílvia Caldeira, Patrícia Pontífice Sousa, Sara Pinto, and Beth Rodgers.

## CONFLICT OF INTEREST

The authors declare no conflict of interest.

## Supporting information

Supplementary information.

Supplementary information.

Supplementary information.

## Data Availability

The data that support the findings of this study are available from the corresponding author upon reasonable request.
