# Supplementary material for: Spiritual comfort, spiritual support, and spiritual care: A simultaneous concept analysis
Source: Nurs Forum. 2022 Nov 30;57(6):1559–66. doi: 10.1111/nuf.12845 (PMC10099816; doi:10.1111/nuf.12845)
Supplement: Supplementary file 1 — Supplementary information. [file NUF-57-1559-s003.docx]

**APPENDIX A** - Prisma flow of each individual concept.

Spiritual Comfort

Records identified through database searching (*n* = 34)

Records after duplicates removed (*n* =25)

Records screened (*n* =25)

## Identification

## Screening

## Included

## Eligibility

Records excluded (*n* =12)

- Only abstract available (n= 6)
- No include spiritual comfort (n=6)

Studies included in the review (*n* =4)

Full-text articles excluded (*n* =9)

- No include spiritual comfort concept (n=9)

Full-text articles assessed for eligibility (*n* =13)

**Figure 1** - Prisma flow of the spiritual comfort concept.

Spiritual Support

Records identified through database searching (*n* = 317)

Records after duplicates removed (*n* =201)

Records screened (*n* =201)

## Identification

## Screening

## Included

## Eligibility

Records excluded (*n* =139)

- Only abstract avaliable (n= 35)
- Language (n=11)
- No includes spiritual support (n= 93)

Studies included in the review (*n* =27)

Full-text articles excluded (*n* =35)

- No includes spiritual support concept (n=35)

Full-text articles assessed for eligibility (*n* =62)

**Figure 2** - Prisma flow of the spiritual support concept.

**Figure 3** - Prisma flow of the spiritual care concept.
