# Supplementary material for: Spiritual comfort, spiritual support, and spiritual care: A simultaneous concept analysis
Source: Nurs Forum. 2022 Nov 30;57(6):1559–66. doi: 10.1111/nuf.12845 (PMC10099816; doi:10.1111/nuf.12845)
Supplement: Supplementary file 2 — Supplementary information. [file NUF-57-1559-s001.docx]

**APPENDIX B** - Full detail of SCA with all included studies.

**Table 6** Process model of spiritual comfort with references of included studies.

| Antecedents | References | |
| --- | --- | --- |
| Awareness of religious and spiritual needs | Béphage (2009); Ponte et al (2012) | |
| Emotional support | Béphage (2009); Pinto et al (2016) | |
| Holistic care | Béphage (2009); Pinto et al (2016) | |
| No unresolved (or unfinish) issues | Rice (2015) | |
| Effective Communication | Béphage (2009); Ponte et al (2012); Rice (2015) | |
| Pain and symptom control | Béphage (2009); Ponte et al (2012); Rice (2015) | |
| Patient-centered care | Béphage (2009); Pinto et al (2016); Ponte et al (2012) | |
| Patient-healthcare team relationship | Béphage (2009); Ponte et al (2012); Rice (2015) | |
| Prevention of social isolation | Béphage (2009); | |
| Spiritual and religious beliefs | Béphage (2009); Pinto et al (2016); Ponte et al (2012) | |
| Attributes | **References** | |
| Connection with self, others, environment, Superior or God | Ponte et al (2012) | |
| Perception of the patient | Ponte et al (2012); Rice (2015) | |
| Gratitude to God | Ponte et al (2012) | |
| Immediate state | Pinto et al (2016) | |
| Outcome | Pinto et al (2016) | |
| Spiritual and religious practices | Béphage (2009); Pinto et al (2016); Ponte et al (2012) | |
| Transcendence | Pinto et al (2016); Ponte et al (2012) | |
| Nurturing environment | Pinto et al (2016); Ponte et al (2012) | |
| Outcomes | **References** |  |
| Sense of inner peace | Pinto et al (2016) |  |
| Wellbeing | Béphage (2009); Pinto et al (2016); Ponte et al (2012) |  |
| Feeling supported by family and healthcare professionals | Pinto et al (2016); Ponte et al (2012) |  |

**Table 7** Process model of spiritual support with references of included studies.

| Antecedents | References |
| --- | --- |
| Awareness of needs of the patient | Bakiler & Eksi (2018); Balboni et al. (2007); Balboni et al. (2011); Balboni et al. (2013); Caixeta et al. (2012); Jones (2020); Kisvetrová et al. (2013); Kuuppelomaèki (2001); Levine et al. (2015); Maton (1989); Praities (2011); Roff et al. (2009); Saad &Medeiros (2016); Rosenbaum et al. (2011); Silva et al. (2019); Strang & Strang (2006). |
| Awareness of religious or spiritual resources | Bakiler & Eksi (2018); Balboni et al. (2007); Balboni et al. (2011); Best et al. (2013); Brandt et al. (2012); Caixeta et al. (2012); Jones (2020); Kisvetrová et al. (2013); Kuuppelomaèki (2001); Meluch, (2018); Roff et al. (2009); Saad &Medeiros (2016); Saad &Medeiros (2016); Soroka et al. (2018); Strang & Strang (2006); Silva et al. (2019). |
| Effective relationship and communication between patient and health care team | Bakiler & Eksi (2018); Balboni et al. (2011); Balboni, et al. (2013); Best et al. (2013); Jones (2020); Kisvetrová et al. (2013); Kuuppelomaèki (2001); Levine et al. (2015); Maton (1989); Silva et al. (2019). |
| Holistic Approach | Balboni et al. (2007); Balboni et al. (2011); Balboni et al. (2013); Best et al. (2013); Brandt et al. (2012); Guilherme et al. (2016) Jones (2020); Roff et al. (2009); Rosenbaum et al. (2011); Soroka et al. (2018) Strang & Strang (2006); Silva et al. (2019); Wilks et al. (2013); Wright et al. (1985). |
| One’s spiritual and religious beliefs | Ai et al. (2005); Bakiler & Eksi (2018); Balboni et al. (2007); Balboni et al. (2011); Balboni et al. (2013); Best et al. (2013); Brandt et al. (2012); Caixeta et al. (2012); Guilherme et al. (2016); Jones (2020); Kisvetrová et al. (2013); Levine et al. (2015); Maton (1989); Meluch, (2018); Nelson-Becker (2005); Ragsdale et al. (2014); Roff et al. (2009); Saad &Medeiros (2016); Silva et al. (2019). |
| Sensitive and knowledgeable in assessing a individual’s religious, spiritual and cultural beliefs, and value systems | Bakiler & Eksi (2018); Balboni et al. (2007); Balboni et al. (2011); Best et al. (2013); Brandt et al. (2012); Caixeta et al. (2012); Jones (2020); Kisvetrová et al. (2013); Levine et al. (2015); Maton (1989); Roff et al. (2009); Rosenbaum et al. (2011); Saad &Medeiros (2016); Kuuppelomaèki (2001); 019); Strang & Strang (2006); Silva et al. (2019) |
| Share decision-making | Best et al. (2013) |
| Attributes | **References** |
| Advanced Care Planning | Balboni et al. (2007); Balboni et al. (2013). |
| Closeness to the sacred or transcendent | Ai et al. (2005); Balboni, et al. (2007); Best et al. (2013); Brandt et al. (2012); Guilherme et al. (2016); Kuuppelomaèki (2001); Maton (1989); Meluch (2018); Nelson-Becker (2005); Ragsdale et al. (2014); Rahmawati et al. (2018); Roff et al. (2009); Rosenbaum et al. (2011); Strang & Strang (2006). |
| Connection with God, members of religious communities, family members and friends, and health care professionals | Ai et al. (2005); Best et al. (2013); Balboni, et al. (2007); Balboni, et al. (2007); Caixeta et al. (2012); Jones (2020); Kisvetrová et al. (2013); Kuuppelomaèki (2001); Maton (1989); Meluch (2018); Nelson-Becker (2005) et al. (2014); Rahmawati et al. (2018); Ragsdale et al. (2014); Roff et al. (2009); Rosenbaum et al. (2011); Strang & Strang (2006). |
| Ethics | Balboni, et al. (2007) Balboni, et al. (2013) Jones (2020); Kisvetrová et al. (2013); Silva et al. (2019). |
| Intimate and affectionate relationship with higher power or God | Brandt et al. (2012); Jones (2020); Kuuppelomaèki (2001); Maton (1989); Nelson-Becker (2005); Ragsdale et al. (2014); Rahmawati et al. (2018); Rosenbaum et al. (2011); Strang & Strang (2006). |
| Intrapersonal Phenomenon | Ai et al. (2005); Brandt et al. (2012); Wilks et al. (2013). |
| Perceived positive influence | Ai et al. (2005); Bakiler & Eksi (2018); Meluch (2018); Nelson-Becker (2005); Ragsdale et al. (2014); Rahmawati et al. (2018); Roff et al. (2009); Rosenbaum et al. (2011); Strang & Strang (2006); Wright et al. (1985); Silva et al. (2019) |
| Perceived social–environmental resource | Jones (2020); Kisvetrová et al. (2013); Kuuppelomaèki (2001); Levine et al. (2015); Meluch (2018); Nelson-Becker (2005); Roff et al. (2009); Rosenbaum et al. (2011); Strang & Strang (2006), |
| Personal supportive components of an individual’s relationship with God | Ai et al. (2005); Brandt et al. (2012); Best et al. (2013); Jones (2020); Kisvetrová et al. (2013); Kuuppelomaèki (2001); Maton (1989); Meluch (2018); Nelson-Becker (2005); Ragsdale et al. (2014); Rahmawati et al. (2018); Roff et al. (2009); Rosenbaum et al. (2011); Strang & Strang (2006). |
| Process which changes the perception of life | Bakiler & Eksi (2018); Jones (2020); Nelson-Becker (2005); Ragsdale et al. (2014); Rosenbaum et al. (2011); Strang & Strang (2006) |
| Therapeutic nature | Guilherme et al. (2016); Kisvetrová et al. (2013); Nelson-Becker (2005); Rahmawati et al. (2018); Ragsdale et al. (2014); Strang & Strang (2006). |
| Outcomes | **References** |
| Comfort | Ai et al. (2005); Balboni et al. (2011); Balboni et al. (2013); Balboni et al. (2007); Caixeta et al. (2012); Guilherme et al. (2016); Jones (2020). |
| Coping with illness | Ai et al. (2005); Balboni et al. (2011); Balboni et al. (2013); Balboni et al. (2007); Best et al. (2013); Brandt et al. (2012); Caixeta et al. (2012); Levine et al. (2015); Maton (1989); Roff et al. (2009); Wilks et al. (2013); Wright et al. (1985) |
| Face life as gift of God | Bakiler & Eksi (2018). |
| Improve Mental Health | Ai et al. (2005); Brandt et al. (2012); Best et al. (2013); Caixeta et al. (2012); Guilherme et al. (2016); Jones (2020); Levine et al. (2015); Maton (1989); Rahmawati et al. (2018) |
| Improving sense of self | Ai et al. (2005); Bakiler & Eksi (2018); Balboni et al. (2013); Balboni et al. (2007); Best et al. (2013); Brandt et al. (2012); Caixeta et al. (2012); Guilherme et al. (2016); Rosenbaum et al. (2011). |
| Meaning and finding purpose | Ai et al. (2005); Bakiler & Eksi (2018); Balboni et al. (2007); Balboni et al. (2011); Balboni et al. (2013); Best et al. (2013); Brandt et al. (2012); Guilherme et al. (2016); Jones (2020); Meluch (2018). |
| Perceived support from God | Ai et al. (2005); Bakiler & Eksi (2018); Guilherme et al. (2016); Roff et al. (2009); Rosenbaum et al. (2011). |
| Positive impact on the healing process | Balboni et al. (2007); Balboni et al. (2011); Balboni et al. (2013); Brandt et al. (2012); Caixeta et al. (2012); Ragsdale et al. (2014); Rahmawati et al. (2018); Roff et al. (2009); Saad & Medeiros (2016); Silva et al. (2019). |
| Quality of life | Balboni et al. (2011); Balboni et al. (2013); Balboni et al. (2007); Best et al. (2013); Brandt et al. (2012); Caixeta et al. (2012); Levine et al. (2015). |
| Reinforcing core beliefs and value systems | Bakiler & Eksi (2018); Balboni et al. (2007); Balboni, et al. (2013); Caixeta et al. (2012); Ragsdale et al. (2014); Rosenbaum et al. (2011). |
| Resilience | Ai et al. (2005); Wilks et al. (2018) |
| Satisfaction with care | Balboni et al. (2011); Best et al. (2013). |
| Self-esteem | Ai et al. (2005); Bakiler & Eksi (2018); Balboni et al. (2013); Brandt et al. (2012); Caixeta et al. (2012); Guilherme et al. (2016); Kisvetrová et al. (2013); Maton (1989). |
| Sense of security | Caixeta et al. (2012). |
| Social cohesiveness | Best et al. (2013); Brandt et al. (2012); Guilherme et al. (2016) |
| Spiritual transformation | Best et al. (2013); Brandt et al. (2012); Caixeta et al. (2012); Rosenbaum et al. (2011); Strang & Strang (2006). |
| Terminal illness awareness | Bakiler & Eksi (2018); Balboni et al. (2013); Best et al. (2013); Kisvetrová et al. (2013); Kuuppelomaèki (2001); Rahmawati et al. (2018). |
| Wellbeing | Balboni et al. (2013); Balboni et al. (2007); Best et al. (2013); Brandt et al. (2012); Caixeta et al. (2012); Guilherme et al. (2016); Jones (2020); Kisvetrová et al. (2013); Levine et al. (2015); Maton (1989); Saad & Medeiros (2016) |

**Table 8** Process model of spiritual care with references of included studies.

| Antecedents | References | |
| --- | --- | --- |
| Identification and assessment of spiritual needs | Moosavi et al (2020); Tüzer et al (2020); O’Callaghan et al (2019); Green & Kim-Godwin (2019); Pipkins et al (2019); Chung et al (2007); Martins et al (2015); McBrien (2010); Rushton (2014); Melhem et al (2016); Dover & Bacon (2001); Gebhardt (2008); Hubel et al (2006); Lundberg & Kerdonfag (2009); Lundmark (2006); Marzband et al (2016); McBrien (2010); Milligan (2011); Ozbasaran et al (2011); Pesut (2006); Pilaikiat et al (2016); Ramezani et al (2014); Ross (2006); Ruder (2013); Sanders et al (2016); Sinclair et al (2012); Stephenson & Wilson (2004); Stranahan (2001); Timmins & Caldeira (2017); Toker & Çınar (2018); Leeuwen & Cusveller (2004); Leeuwen et al (2006); Leeuwen et al (2009); Gómez et al (2016); Walker & Waterworth (2017); Wong et al (2008); Atarhim et al (2019); Battey (2012); Biley & Freshwater (1998); Burkhart & Hogan (2008); Carroll (2001); Carson (2011); Cavendish et al (2006); Musgrave & McFarlane (2003); Deal (2008); DeKoninck et al (2016); Dhamani et al (2011); Baldacchino (2006); Baldacchino (2008); Green et al (2020); Wright (1998); Kevern (2012); Kudubes et al (2019) | |
| Intentionality | Marzband et al (2016); Ramezani et al (2014) | |
| Moral skills | Moosavi et al (2020); Tüzer et al (2020); Pilaikiat et al (2016); Ramezani et al (2014) | |
| Multidisciplinary team approach | Moosavi et al (2020); Melhem et al (2016); Lundberg & Kerdonfag (2009); Milligan (2011); Ruder (2013); Tiew et al (2012); Leeuwen & Cusveller (2004); | |
| Perception of spiritual care | Chung et al (2007); Melhem et al (2016); Ramezani et al (2014); Stranahan (2001); Gómez et al (2016); Atarhim et al (2019); Azarsa et al (2015); Musgrave & McFarlane (2003); Chan (2010); Chan et al (2006); Cooper et al (2020); Ebrahimi et al (2017); Kaddourah et al (2018) | |
| Professional commitment | Ramezani et al (2014); Gómez et al (2016); Wong et al (2008); Lundberg & Kerdonfag (2009); Ce (1998); Connerton & Moe (2018); Kevern (2012); | |
| Religious/spiritual rituals | Moosavi et al (2020); Tüzer et al (2020); O’Callaghan et al (2019); Green & Kim-Godwin (2019); Pipkins et al (2019); Chung et al (2007); McBrien (2010); Melhem et al (2016); Silva et al (2015); Dover & Bacon (2001); Gebhardt (2008); Hubel et al (2006); Lundmark (2006); McBrien (2010); Musa & Pevalin (2015); Pilaikiat et al (2016); Ruder (2013); Sanders et al (2016); Stephenson & Wilson (2004); Stranahan (2001); Tirgari et al (2013); Leeuwen & Cusveller (2004); Gómez et al (2016); Walker & Waterworth (2017); Battey (2012); Bowers & Rieg (2014); Burkhart & Hogan (2008); Caldeira & Timmins (2017); Carroll (2001); Carson (2011); Cooper et al (2020); Baldacchino (2008); Ku (2017) | |
| Respecting and supporting spiritual/religious beliefs | Moosavi et al (2020); Tüzer et al (2020); O’Callaghan et al (2019); Green & Kim-Godwin (2019); Pipkins et al (2019); Chung et al (2007); McBrien (2010); Melhem et al (2016); Silva et al (2015); Dover & Bacon (2001); Gebhardt (2008); Hubel et al (2006); Lundberg & Kerdonfag (2009); Lundmark (2006); McBrien (2010); Musa & Pevalin (2015); Ozbasaran et al (2011); Pilaikiat et al (2016); Ruder (2013); Sanders et al (2016); Stephenson & Wilson (2004); Stranahan (2001); Tirgari et al (2013); Leeuwen & Cusveller (2004); Gómez et al (2016); Walker & Waterworth (2017); Wong et al (2008); Caldeira & Timmins (2017); Carson (2011); Ebrahimi et al (2017); Green et al (2020); | |
| Sensitivity concerning spiritual care | Moosavi et al (2020); Tüzer et al (2020); O’Callaghan et al (2019); Green & Kim-Godwin (2019); Pipkins et al (2019); Chung et al (2007); Martins et al (2015); McBrien (2010); Rushton (2014); Melhem et al (2016); Dover & Bacon (2001); Gebhardt (2008); Hubel et al (2006); Lundberg & Kerdonfag (2009); Lundmark (2006); Marzband et al (2016); McBrien (2010); Milligan (2011); Ozbasaran et al (2011); Pesut (2006); Pilaikiat et al (2016); Ramezani et al (2014); Ross (2006); Ruder (2013); Sanders et al (2016); Sinclair et al (2012); Stephenson & Wilson (2004); Stranahan (2001); Timmins & Caldeira (2017); Toker & Çınar (2018); Leeuwen & Cusveller (2004); Leeuwen et al (2006); Leeuwen et al (2009); Gómez et al (2016); Walker & Waterworth (2017); Wong et al (2008); Atarhim et al (2019); Battey (2012); Biley & Freshwater (1998); Burkhart & Hogan (2008); Carroll (2001); Carson (2011); Cavendish et al (2006); Musgrave & McFarlane (2003); Deal (2008); DeKoninck et al (2016); Dhamani et al (2011); Baldacchino (2006); Baldacchino (2008); Green et al (2020); Wright (1998); Kevern (2012); Kudubes et al (2019) | |
| Spiritual/transcendent self-awareness (nurse and patient) | Moosavi et al (2020); Tüzer et al (2020); Green & Kim-Godwin (2019); Pipkins et al (2019); Chung et al (2007); McBrien (2010); Melhem et al (2016); Dover & Bacon (2001); Gebhardt (2008); Hubel et al (2006); Lundmark (2006); Marzband et al (2016); Musa & Pevalin (2015); Pilaikiat et al (2016); Polzer et al (2012); Ramezani et al (2014); Ross (2006); Ross et al (2013); Ruder (2013); Sanders et al (2016); Sawatzky & Pesut (2005); Stranahan (2001); Timmins & Caldeira (2017); Toker & Çınar (2018); Leeuwen & Cusveller (2004); Leeuwen et al (2006); Leeuwen et al (2009); Gómez et al (2016); Vincensi & Solberg (2017); Wim Smeets et al (2020); Akgün et al (2016); Atarhim et al (2019); Azarsa et al (2015); Battey (2012); Biley & Freshwater (1998); Burkhart & Hogan (2008); Caldeira & Timmins (2017); Carroll (2001); Carson (2011); Cavendish et al (2006); Musgrave & McFarlane (2003); Musgrave & McFarlane (2004); Chan (2010); Chan et al (2006); Chandramohan & Bhagwan (2016); Meehan (2012); Connerton & Moe (2018); Cooper et al (2020); Baldacchino (2006); Plessis (2016); Govier (2000); Green et al (2020); Kaddourah et al (2018); Wright (1998); Kociszewski (2003); Ku (2017); Kudubes et al (2019) | |
| Therapeutic communication | Moosavi et al (2020); Green & Kim-Godwin (2019); Melhem et al (2016); McBrien (2010); Milligan (2011); Ramezani et al (2014); Ross (2006); Leeuwen et al (2013); Sanders et al (2016); Sawatzky & Pesut (2005); Leeuwen & Cusveller (2004); Leeuwen et al (2006); Willemse et al (2020); Caldeira & Timmins (2017); Connerton & Moe (2018); Dhamani (2011); Baldacchino (2006); Baldacchino (2008); Ebrahimi et al (2017); Green et al (2020); Wright (1998); | |
| Trust relationship between nurse patient | Moosavi et al (2020); Martins et al (2016); McBrien (2010); Rushton (2014); Melhem et al (2016); Gebhardt (2008); Hubel et al (2006); Lundberg & Kerdonfag (2009); Bowers & Rieg (2014); Carroll (2001); Ce (1998); Connerton & Moe (2018); Deal (2008); Baldacchino (2006); Baldacchino (2008); Plessis (2016); Ebenau et al (2020); Kh (2008); | |
| Attributes | **References** |  |
| Altruistic | Velásquez & Gómez (2014); Lundberg & Kerdonfag (2009); Milligan (2011); Ferrel & Munevar (2012); |  |
| Comfortable, therapeutic, nurturing and healing environment | Ramezani et al (2014); Veloza-Gómez et al (2016); Wong & Yau (2008); Caldeira & Timmins (2017); Connerton & Moe (2018); Christensen & Turner (2008); Kociszewski (2004); |  |
| Compassion and dignified care | O’Callaghan et al (2019); McBrien (2010); Milligan (2011); Leeuwen et al (2013); Sawatzky & Pesut (2005); Timmins & Caldeira (2017); Veloza-Gómez et al (2016); Bone et al (2018); Meehan (2012); Ferrel & Munevar (2012); Greasley & Gartland (2001); Kroning (2017); Velásquez & Gómez (2014) |  |
| Complex | Ramezani et al (2014); Baldacchino (2006); Christensen & Turner (2008); Kociszewski (2004); Govier (2000) |  |
| Connection with self, others, and/or God or Superior being | O’Callaghan et al (2019); Gebhardt (2008); Ramezani et al (2014); Wong & Yau (2008); Atarhim et al (2019); Babamohamadi et al (2020); Burkhart & Hogan (2008); Carson (2011); Cavendish et al (2006); Ce (1998); Deal (2008); Plessis (2016); Kociszewski (2004); Kroning (2017) |  |
| Fostering the search for meaning | Vogel & Schep-Akkerman (2018); Marzband et al (2016); Ramezani et al (2014); Ross et al (2013); Sawatzky & Pesut (2005); Sinclair et al (2012); Stephenson & Wilson (2004); Timmins & Caldeira (2017); Leeuwen et al (2009); Vincensi & Burkhart (2014); Walker & Waterworth (2017); Willemse et al (2020); Wong & Yau (2008); Cavendish et al (2006); Musgrave & McFarlane (2003); Chan (2010); Chandramohan & Bhagwan (2016); Cooper et al (2020); Deal (2008); Baldacchino (2008); Plessis (2016); Egan et al (2017); Greasley & Gartland (2001); Herlianita et al (2018); Wright (1998) |  |
| Harmonious connectedness | Stephenson & Wilson (2004); Willemse et al (2020); O’Callaghan et al (2019); Baldacchino (2008); Plessis (2016); Kociszewski (2004); Burkhart & Hogan (2008); Cavendish et al (2006); |  |
| Holistic and patient and patient-centered care | Pipkins et al (2019); Chung et al (2007); Rushton (2014); Moosavi et al (2020); Ozbasaran et al (2011); Tiew et al (2012); Tirgari et al (2013); Dhamani et al (2011); Vincensi & Burkhart (2014); |  |
| Interactive and intentional process | Silva et al (2015); Lundberg & Kerdonfag (2009); Marzband et al (2016); Milligan (2011); Pilaikiat et al (2016); Ramezani et al (2014); Stephenson & Wilson (2004); Bone et al (2018) |  |
| Interpersonal | Velásquez & Gómez (2014); Lundberg & Kerdonfag (2009); Marzband et al (2016); Milligan (2011); Ramezani et al (2014); Ruder (2013); Chan (2010); Meehan (2012); Egan et al (2017); Ferrel & Munevar (2012); Greasley & Gartland (2001) |  |
| Intimate | Chandramohan & Bhagwan (2016); Deal (2008); Fowler (2020); |  |
| Intuitive sense | Lundberg & Kerdonfag (2009); Milligan (2011); Ramezani et al (2014); Ruder (2013); Caldeira & Timmins (2017); Ferrel & Munevar (2012) |  |
| Is an ethic | Milligan (2011); Pesut (2006) |  |
| Just being there | Carroll (2001) |  |
| Mostly subjective | Govier (2000) |  |
| Multifaceted phenomenon; | Carroll (2001) |  |
| Nursing spiritual care intervention | Green & Kim-Godwin (2019); Lundmark (2006); Marzband et al (2016); Ramezani et al (2014); Sawatzky & Pesut (2005); Ku (2017); Burkhart & Hogan (2008); Caldeira & Timmins (2017); Cavendish et al (2006); Ce (1998); Musgrave & McFarlane (2003); Chandramohan & Bhagwan (2016); Dhamani et al (2011); Baldacchino (2008); Green et al (2020); Herlianita et al (2018); Kevern (2012); Christensen & Turner (2008); |  |
| Personal | Cooper (2020); Glasper (2011); Greasley & Gartland (2001); Kociszewski (2004) |  |
| Presence | Green & Kim-Godwin (2019); McBrien (2010); Marzband et al (2016); Sawatzky & Pesut (2005); Moosavi et al (2020); Bone et al (2018); Caldeira & Timmins (2017); Carson (2011); Plessis (2016); Ferrel & Munevar (2012); Glasper (2011) |  |
| Therapeutic environment | Kociszewski (2004); |  |
| Therapeutic nature | Ramezani et al (2014); Sawatzky & Pesut (2005); Veloza-Gómez et al (2016); Biley & Freshwater (1998); Caldeira & Timmins (2017); Connell (2012); Connerton & Moe (2018); Baldacchino (2008); Egan et al (2017); Taylor (2008); Green et al (2020); Wright (1998); Kevern (2012); Christensen & Turner (2008); Kociszewski (2004) |  |
| Therapeutic use of self | Ramezani et al (2014); Sawatzky & Pesut (2005); Caldeira & Timmins (2017); Baldacchino (2008) |  |
| Transcendence | Moosavi et al (2020); Lundberg & Kerdonfag (2009); Milligan (2011); Deal (2008); Ferrel & Munevar (2012); |  |
| Outcomes | **References** |  |
| Adherence of treatments | Moosavi et al (2020); Helming (2009) |  |
| Appreciation | Moosavi et al (2020) |  |
| Comfort | Melhem et al (2016); Silva et al (2015); Noormohammadi et al (2019); Dover & Bacon (2001); Gebhardt (2008); Musa & Pevalin (2015); Sawatzky & Pesut (2005); Stephenson & Wilson (2004); Walker & Waterworth (2017); Willemse et al (2020); Wong & Yau (2008); Deal (2008); Dhamani et al (2011); Kroning (2017); |  |
| Cooperation | Chandramohan & Bhagwan (2016); Connerton & Moe (2018); Helming (2009) |  |
| Coping | Noormohammadi et al (2019); Gebhardt (2008); Hubel et al (2006); Lundberg & Kerdonfag (2009); Marzband et al (2016); Musa & Pevalin (2015); Pilaikiat et al (2016); Ramezani et al (2014); Ruder (2013); ); Timmins & Caldeira (2017); Tirgari et al (2013); Chandramohan & Bhagwan (2016); Connerton & Moe (2018); Kroning (2017) |  |
| Express feelings of love, humility, and gratitude E | Sawatzky & Pesut (2005); Tirgari et al (2013); Kociszewski (2004); Kroning (2017) |  |
| Gratefulness | Musa & Pevalin (2015); Moosavi et al (2020) |  |
| Healing | Dover & Bacon (2001); Marzband et al (2016); Milligan (2011); Ramezani et al (2014); Biley & Freshwater (1998); Chandramohan & Bhagwan (2016); Connerton & Moe (2018); Helming (2009) |  |
| Holistic health of patients | Chan et al (2006) |  |
| Hope | O’Callaghan et al (2019); McBrien (2010); Melhem et al (2016); Noormohammadi et al (2019); Marzband et al (2016); Casarez & Engebretson (2012); Ramezani et al (2014); Tiew et al (2012); Tirgari et al (2013); Wong et al (2008); Moosavi et al (2019) |  |
| Improve relationship with himself, others, God, and nature | Moosavi et al (2020); Hubel et al (2006); Gallison et al (2013); Kociszewski (2004); Kroning (2017) |  |
| Inner peace | Musa & Pevalin (2015); Lundberg & Kerdonfag (2009); Ramezani et al (2014); Atarhim et al (2019); Connerton & Moe (2018) |  |
| Nurses feel bonused/nurse satisfaction | Gebhardt (2008); Gallison et al (2013); Kociszewski (2004) |  |
| Pain management | Melhem et al (2016); Musa & Pevalin (2015); Pilaikiat et al (2016); Tirgari et al (2013); Wong & Yau (2008); Connerton & Moe (2018); |  |
| Physical relaxation, forgiveness of others | Musa & Pevalin (2015); Sawatzky & Pesut (2005); Tirgari et al (2013); Connerton & Moe (2018); |  |
| Positive emotions | Moosavi et al (2020); O’Callaghan et al (2019); Chung et al (2007); Martins et al (2015); McBrien (2010); Velásquez & Gómez (2014); Melhem et al (2016); Silva et al (2015); Noormohammadi et al (2019); Lundberg & Kerdonfag (2009); Marzband et al (2016); Musa & Pevalin (2015); Ramezani et al (2014); Stephenson & Wilson (2004); Tirgari et al (2013); Wong & Yau (2008); Burkhart & Hogan (2008) |  |
| Quality of care | Chan et al (2006); Veloza-Gómez et al (2016); |  |
| Quality of life | Moosavi et al (2020); Melhem et al (2016); Marzband et al (2016); Pilaikiat et al (2016); Willemse et al (2020) |  |
| Reduced depression | Green & Kim-Godwin (2019); Melhem et al (2016); Marzband et al (2016); Pilaikiat et al (2016); Ramezani et al (2014) |  |
| Spiritual growth | Hubel et al (2006); Ramezani et al (2014); Connerton & Moe (2018); Baldacchino (2008) |  |
| Spiritual health | Noormohammadi et al (2019); Babamohamadi et al (2020); Chan et al (2006); Plessis (2016) |  |
| Spiritual integrity | Ramezani et al (2014); Noormohammadi et al (2019); Plessis (2016) |  |
| Spiritual wellbeing/ weelbeing | Moosavi et al (2020); O’Callaghan et al (2019); Pipkins et al (2019); Chung et al (2007); Vogel & Schep-Akkerman (2018); Velásquez & Gómez (2014); Rushton (2014); Hubel et al (2006); McBrien (2010); Pesut (2006); Casarez & Engebretson (2012); Ramezani et al (2014); Ross (2006); Leeuwen et al (2013); Ruder (2013); Sanders et al (2016); Sinclair et al (2012); Timmins & Caldeira (2017); Tirgari et al (2013); Veloza-Gómez et al (2016); Vicensi & Solberg (2017); Walker & Waterworth (2017); Willemse et al (2020); Wong et al (2008); ); Azarsa et al (2015); Biley & Freshwater (1998); Ce (1998); Musgrave & McFarlane (2003); Plessis (2016) |  |
| Willingness/readiness | Moosavi et al (2020) |  |

**APPENDIX C – Consulted references for each concept**

**Spiritual Comfort references**

Béphage, G. (2009). Promoting spiritual comfort in palliative care settings. Nursing & Residential Care, 11(9), 463-466. doi: 10.12968/nrec.2009.11.9.43732

Pinto, S., Berenguer, S., Martins, J., Kolcaba. K. (2016a). Cultural adaptation and validation of the Portuguese End of Life Spiritual Comfort Questionnaire in Palliative Care patients. Porto Biomedial Journal, 1(4), 147-152. doi: 10.1016/j.pbj.2016.08.003

Ponte, K., Silva, L., Aragão, A., Guedes, M., Zagonel, I. (2012). Contribuição do cuidado clínico de enfermagem para o conforto psicoespiritual de mulheres com infarto agudo do miocárdio. Escola Anna Nery, 16(4), 666-673. doi: 10.1590/S1414-81452012000400004

Rice, L. (2015). The spiritual comfort measure. Journal of palliative medicine, 18(11), 910-911. doi: 10.1089/jpm.2015.0262

**Spiritual support references**

Ai, A. L., Tice, T. N., Peterson, C., & Huang, B. (2005). Prayers, spiritual support, and positive attitudes in coping with the September 11 national crisis. *Journal of personality, 73*(3), 763-791.

Bakiler, E., & Eksi, H. (2018). A Phenomenological Analysis of the Spiritual Support Services in State Hospitals: The Case of Turkey. *The journal of pastoral care & counseling : JPCC, 72*(2), 116-128. doi:10.1177/1542305018766277

Balboni, M. J., Babar, A., Dillinger, J., Phelps, A. C., George, E., Block, S. D., . . . Balboni, T. A. (2011). “It Depends”: Viewpoints of Patients, Physicians, and Nurses on Patient-Practitioner Prayer in the Setting of Advanced Cancer. *Journal of Pain and Symptom Management, 41*(5), 836-847. doi: 10.1016/j.jpainsymman.2010.07.008

Balboni, T. A., Balboni, M., Enzinger, A. C., Gallivan, K., Paulk, M. E., Wright, A., . . . Prigerson, H. G. (2013). Provision of spiritual support to patients with advanced cancer by religious communities and associations with medical care at the end of life. *JAMA Internal Medicine, 173*(12), 1109-1117. doi:10.1001/jamainternmed.2013.903

Balboni, T. A., Vanderwerker, L. C., Block, S. D., Paulk, M. E., Lathan, C. S., Peteet, J. R., & Prigerson, H. G. (2007). Religiousness and spiritual support among advanced cancer patients and associations with end-of-life treatment preferences and quality of life. *Journal of clinical oncology : official journal of the American Society of Clinical Oncology, 25*(5), 555-560.

Best, M., Butow, P., Olver, I. (2013). Spiritual Support of cancer patients and the role of the doctor. *Support Care Cancer, 22(5)*, 1333-1339. doi: 10.1007/s00520-013-2091-1

Brandt, P.-Y., Mohr, S., Gillièron, C., Rieben, I., & Huguelet, P. (2012). Religious Coping in Schizophrenia Patients: Spiritual Support in Medical Care and Pastoral Counselling. *Toronto Journal of Theology, 28*(2), 193-208.

Caixeta, C. R. d. C. B., Nascimento, L. C., Pedro, I. C. d. S., & Rocha, S. M. M. (2012). Spiritual support for people living with HIV/ AIDS: A Brazilian explorative, descriptive study. *Nursing & Health Sciences, 14*(4), 514-519.

Guilherme, C., Ribeiro, G. R., Zamarioli, C. M., Almeida, A. M., de Carvalho, E. C., Caldeira, S., & de Souza Oliveira-Kumakura, A. R. (2016). Effect of the "Spiritual Support" Intervention on Spirituality and the Clinical Parameters of Women Who Have Undergone Mastectomy: A Pilot Study. *Religions, 7*(3), 26-26.

Jones, C. (2020). Where do critical care relatives turn for spiritual support and comfort? *Intensive & critical care nursing, 58*, 102817-102817. doi:10.1016/j.iccn.2020.102817

Kisvetrová, H., Klugar, M., & Kabelka, L. (2013). Spiritual support interventions in nursing care for patients suffering death anxiety in the final phase of life. *International Journal of Palliative Nursing, 19*(12), 599-605.

Kuuppelomäki, M. (2001). Spiritual support for terminally ill patients: nursing staff assessments. *Journal of Clinical Nursing (Wiley-Blackwell), 10*(5), 660-670. doi:10.1046/j.1365-2702.2001.00534.x

Levine, E., Vong, S., & Yoo, G. (2015). Development and Initial Validation of a Spiritual Support Subscale for the MOS Social Support Survey. *Journal of Religion & Health, 54*(6), 2355-2366. doi:10.1007/s10943-015-0005-x

Maton, K. I. (1989). The Stress-Buffering Role of Spiritual Support: Cross-Sectional and Prospective Investigations. *Journal for the Scientific Study of Religion, 28*(3), 310-310.

Meluch, A. L. (2018). Spiritual Support Experienced at a Cancer Wellness Center. *Southern Communication Journal, 83*(3), 137-148.

Nelson-Becker, H. (2005). Development of a spiritual support scale for use with older adults. *Journal of Human Behavior in the Social Environment, 11*(3/4), 195-212.

Rahmawati, I., Wihastuti, T. A., Rachmawati, S. D., Kumboyono, K. (2018). Nursing Experience in Providing Spiritual Support to Patients with Acute Coronary Syndrome at Emergency Unit: Phenomenology Study. International Journal of Caring Sciences, 11(2), 1147-1151. Available at: http://www.internationaljournalofcaringsciences.org/docs/58_kompoyono_original_10_2.pdf

Ragsdale, J. R., Hegner, M. A., Mueller, M., & Davies, S. (2014). Identifying Religious and/or Spiritual Perspectives of Adolescents and Young Adults Receiving Blood and Marrow Transplants: A Prospective Qualitative Study. *Biology of Blood and Marrow Transplantation, 20*(8), 1242-1247. doi:10.1016/j.bbmt.2014.04.013

Roff, L. L., Simon, C. E., Nelson-Gardell, D., & Pleasants, H. M. (2009). Spiritual Support and African American Breast Cancer Survivors. *Affilia: Journal of Women & Social Work, 24*(3), 285-299.

Rosenbaum, J. L., Smith, J. R., & Zollfrank, R. (2011). Neonatal end-of-life spiritual support care. *The Journal of perinatal & neonatal nursing, 25*(1), 61-69. doi:10.1097/JPN.0b013e318209e1d2

Saad, M., & de Medeiros, R. (2016). Programs of religious/spiritual support in hospitals - five "Whies" and five "Hows". *Philosophy, Ethics & Humanities in Medicine, 11*, 1-4.

Silva, T., Freire, M. E., Vasconcelos, M. F. d., Rodrigues, L., Matias, T., & Neto, M. (2019). Apoio espiritual ao paciente hospitalizado: percepções da equipe multiprofissional de saúde TT - Spiritual support for hospitalized patients: perceptions of the multiprofissional health team. *Psicologia, Saúde &amp; Doenças, 20*(2), 358-366. doi:10.15309/19psd200207

Soroka, J., Collins, L., Creech, G., Kutcher, G., Menne, K., & Petzel, B. (2018). Spiritual Care at the End of Life. Does Educational Intervention Focused on a Broad Definition of Spirituality Increase Utilization of Chaplain Spiritual Support in Hospice? *Journal of Pain and Symptom Management, 56*(6), e41-e41. doi:[10.1016/j.jpainsymman.2018.10.018](about:blank)

Strang, S., Strang, P. (2006). Spiritual Support for Palliative Care Patients - a Duty for Hospital Chaplains and/or Health Care Staff ? *Supportive Palliative Cancer Care, 2(2)*, 55-64. Available at: https://www.edisciences.org/scheda-spcc24-vol-2-issue-2

Wilks, S. E., Ketchum, M. M., Chen, Z., Bowman, A. M. (2014). Psychometric Reevaluation of the Spiritual Support Scale in a Sample of Alzheimer's Caregivers. *Journal of Religion & Spirituality in Social Work: Social Thought, 32(4)*, 313-329. doi: 10.1080/15426432.2013.839212

Wilks, S. E., Spurlock, W. R., Brown, S. C., Teegen, B. C., Geiger, J. R. (2018). Examining spiritual support among African American and Caucasian Alzheimer’s caregivers: A risk and resilience study. *Geriatric Nursing, 39(6),* 663-668. doi: 10.1016/j.gerinurse.2018.05.002

Wright, S. D, Pratt, C. C., Schmall, V. L. (1985). Spiritual Support for Caregivers of Dementia Patients. *Journal of Religion and Health, 24(1)*, 31-38. Available at: https://www.jstor.org/stable/27505804?seq=1#metadata_info_tab_contents

**Spiritual care references**

Akgun Sahin, Z., & Kardas Ozdemir, F. (2016). Spirituality and spiritual care: a descriptive survey of nursing practices in Turkey. *Contemp Nurse, 52*(4), 454-461. doi:10.1080/10376178.2016.1221324

Atarhim, M. A., Lee, S., & Copnell, B. (2019). An Exploratory Study of Spirituality and Spiritual Care Among Malaysian Nurses. *J Relig Health, 58*(1), 180-194. doi:10.1007/s10943-018-0624-0

Azarsa, T., Davoodi, A., Khorami Markani, A., Gahramanian, A., & Vargaeei, A. (2015). Spiritual wellbeing, Attitude toward Spiritual Care and its Relationship with Spiritual Care Competence among Critical Care Nurses. *J Caring Sci, 4*(4), 309-320. doi:10.15171/jcs.2015.031

Babamohamadi, H., Kadkhodaei-Elyaderani, H., Ebrahimian, A., & Ghorbani, R. (2020). The Effect of Spiritual Care Based on the Sound Heart Model on the Spiritual Health of Patients with Acute Myocardial Infarction. *Journal of religion and health*. doi:10.1007/s10943-020-01003-w

Baldacchino, D. R. (2006). Nursing competencies for spiritual care. *Journal of Clinical Nursing (Wiley-Blackwell), 15*(7), 885-896. doi:10.1111/j.1365-2702.2006.01643.x

Baldacchino, D. R. (2008). Spiritual care: is it the nurse's role? *Spirituality & Health International, 9*(4), 270-284.

Battey, B. W. (2012). Perspectives of spiritual care for nurse managers. *J Nurs Manag, 20*(8), 1012-1020. doi:10.1111/j.1365-2834.2012.01360.x

Biley, F. C., & Freshwater, D. (1998). Spiritual care and the environment: a new paradigm for nursing? *Complementary therapies in nursing & midwifery, 4*(4), 98-99.

Bone, N., Swinton, M., Hoad, N., Toledo, F., & Cook, D. (2018). Critical Care Nurses' Experiences With Spiritual Care: The SPIRIT Study. *Am J Crit Care, 27*(3), 212-219. doi:10.4037/ajcc2018300

Bowers, H., & Rieg, L. S. (2014). Reflections on Spiritual Care. *Journal of Christian Nursing, 31*(1), 47-51. doi:10.1097/CNJ.0000000000000017

Burkhart, L., & Hogan, N. (2008). An experiential theory of spiritual care in nursing practice. *Qualitative Health Research, 18*(7), 928-938.

Caldeira, S., Pinto, S., & Capelas, M. L. (2017). Implementing spiritual care at the end of life: Portugal. *European Journal of Palliative Care, 24*(4), 175-176.

Caldeira, S., & Timmins, F. (2015). Editorial: Time as presence and opportunity: the key to spiritual care in contemporary nursing practice. *J Clin Nurs, 24*(17-18), 2355-2356. doi:10.1111/jocn.12909

Caldeira, S., & Timmins, F. (2017). Implementing spiritual care interventions. *Nursing Standard, 31*(34), 54-60. doi:10.7748/ns.2017.e10313

Carroll, B. (2001). A phenomenological exploration of the nature of spirituality and spiritual care. *Mortality, 6*(1), 81-98.

Carson, V. B. (2011). What is the essence of spiritual care? *Journal of Christian nursing : a quarterly publication of Nurses Christian Fellowship, 28*(3), 173-173.

Casarez, R. L. P. & Engebretson, J. C. (2012). Ethical issues of incorporating spiritual

care into clinical practice. *Journal of Clinical Nursing, 21*, 2099–2107. doi:10.1111/j.1365-2702.2012.04168.x

Cavendish, R., Konecny, L., Naradovy, L., Bk, L., Como, J., Okumakpeye, P., . . . Lanza, M. (2006). Patients' perceptions of spirituality and the nurse as a spiritual care provider. *Holistic Nursing Practice, 20*(1), 41-47.

Ce, S.-F. (1998). Spiritual care and chronically ill clients. *Home Healthcare Nurse, 16*(4), 243-250.

Chan, M. F. (2010). Factors affecting nursing staff in practising spiritual care. *J Clin Nurs, 19*(15-16), 2128-2136. doi:10.1111/j.1365-2702.2008.02690.x

Chan, M. F., Chung, L. Y., Lee, A. S., Wong, W. K., Lee, G. S., Lau, C. Y., . . . Ng, J. W. (2006). Investigating spiritual care perceptions and practice patterns in Hong Kong nurses: results of a cluster analysis. *Nurse Educ Today, 26*(2), 139-150. doi:10.1016/j.nedt.2005.08.006

Chandramohan, S., & Bhagwan, R. (2016). Utilization of Spirituality and Spiritual Care in Nursing Practice in Public Hospitals in KwaZulu-Natal, South Africa. *Religions, 7*(3). doi:10.3390/rel7030023

Chung, L. Y. F., Wong, F. K. Y., & Chan, M. F. (2007). Relationship of nurses’

spirituality to their understanding and practice of spiritual care. *Journal of Advanced Nursing, 58(2),* 158-170. doi:org/10.1111/j.1365-2648.2007.04225.x

Connell Meehan, T. (2012). Spirituality and spiritual care from a Careful Nursing perspective. *Journal of Nursing Management (John Wiley & Sons, Inc.), 20*(8), 990-1001. doi:10.1111/j.1365-2834.2012.01462.x

Connerton, C. S., & Moe, C. S. (2018). The Essence of Spiritual Care. *Creat Nurs, 24*(1), 36-41. doi:10.1891/1078-4535.24.1.36

Cooper, K. L., Chang, E., Luck, L., & Dixon, K. (2020). How Nurses Understand Spirituality and Spiritual Care: A Critical Synthesis. *Journal of holistic nursing : official journal of the American Holistic Nurses' Association, 38*(1), 114-121. doi:10.1177/0898010119882153

Deal, B. (2008). *The lived experience of giving spiritual care.* Texas Woman's University, Retrieved from http://search.ebscohost.com/login.aspx?direct=true&db=rzh&AN=109851663&lang=pt-pt&site=ehost-live

DeKoninck, B., Hawkins, L. A., Fyke, J. P., Neal, T., & Currier, K. (2016). Spiritual Care Practices of Advanced Practice Nurses: A Multinational Study. *The Journal for Nurse Practitioners, 12*(8), 536-544. doi:10.1016/j.nurpra.2016.05.023

Dhamani, K. A. (2011). *Tanzanian Nurses' Understanding of Spirituality and Practice of Spiritual Care.* University of Alberta (Canada), Retrieved from http://search.ebscohost.com/login.aspx?direct=true&db=rzh&AN=109857225&lang=pt-pt&site=ehost-live

Dover, L. J. Van, & Bacon, J. M. (2001). Spiritual Care in Nursing Practice: A Close-up

View. Nursing Forum, 36(3), 18-28. doi: 10.1111/j.1744-6198.2001.tb00245.x

du Plessis, E. (2016). Presence: A Step Closer to Spiritual Care in Nursing. *Holist Nurs Pract, 30*(1), 47-53. doi:10.1097/HNP.0000000000000124

Ebenau, A., Groot, M., Visser, A., Laarhoven, H. W. M., Leeuwen, R., & Garssen, B. (2020). Spiritual care by nurses in curative oncology: a mixed‐method study on patients' perspectives and experiences. *Scandinavian Journal of Caring Sciences, 34*(1), 96-107. doi:10.1111/scs.12710

Ebrahimi, H., Areshtanab, H. N., Khanmiri, S. G., & Jafarabadi, M. A. (2017). Health Care Providers' Perception of Their Competence in Providing Spiritual Care for Patients. *Indian Journal of Palliative Care, 23*(1), 57-61. doi:10.4103/0973-1075.197957

Egan, R., Llewellyn, R., Cox, B., MacLeod, R., McSherry, W., & Austin, P. (2017). New Zealand Nurses' Perceptions of Spirituality and Spiritual care: Qualitative Findings from a National Survey. *Religions, 8*(5), 79-79.

Ej, T. (2008). What is spiritual care in nursing?: findings from an exercise in content validity. *Holistic Nursing Practice, 22*(3), 154-159.

Ferrell, B., & Munevar, C. (2012). Domain of spiritual care. *Progress in Palliative Care, 20*(2), 66-71. doi:10.1179/1743291X12Y.0000000013

Fowler, M. D. (2020). The Nursing Domain of Spiritual Care. *Journal of Christian nursing : a quarterly publication of Nurses Christian Fellowship, 37*(3), 140-141. doi:10.1097/CNJ.0000000000000727

Gallison, B. S., Xu, Y., Jurgens, C. Y., & Boyle, S. M. (2013). Acute Care Nurses’ Spiritual Care Practices. *Journal of Holistic Nursing, 31*(2), 95-103. doi:10.1177/0898010112464121

Gasper, A. (2011). Can nurses enhance spiritual care in end-of-life settings? *British Journal of Nursing, 20*(5), 316-317.

Gebhardt, M. C. (2008). Rehabilitation nurses’ experiences providing spiritual care.

*Spirituality and Health International, 9*, 230–240. doi:10.1002/shi.353

Govier, I. (2000). Spiritual care in nursing: a systematic approach. *Nursing standard (Royal College of Nursing (Great Britain) : 1987), 14*(17), 32-36.

Greasley, P., Lf, C., & Gartland, M. (2001). The concept of spiritual care in mental health nursing. *Journal of Advanced Nursing (Wiley-Blackwell), 33*(5), 629-637. doi:10.1046/j.1365-2648.2001.01695.x

Green, A., Kim-Godwin, Y. S., & Jones, C. W. (2020). Perceptions of Spiritual Care Education, Competence, and Barriers in Providing Spiritual Care Among Registered Nurses. *Journal of holistic nursing : official journal of the American Holistic Nurses' Association, 38*(1), 41-51. doi:10.1177/0898010119885266

Hanna, K. J. (2018). FAQs in Spiritual Care. Is Self-Esteem Part of Spiritual Care? *Journal of Christian Nursing, 33*(2), 131-131. doi:10.1097/CNJ.0000000000000481

Helming, M. A. (2009). Integrating Spirituality into Nurse Practitioner Practice: The Importance of Finding the Time. *The Journal for Nurse Practitioners, 5*(8), 598-605. doi:https://doi.org/10.1016/j.nurpra.2009.04.012

Herlianita, R., Yen, M., Chen, C. H., Fetzer, S. J., & Lin, E. C. (2018). Perception of Spirituality and Spiritual Care among Muslim Nurses in Indonesia. *J Relig Health, 57*(2), 762-773. doi:10.1007/s10943-017-0437-6

Kaddourah, B., Abu-Shaheen, A., & Al-Tannir, M. (2018). Nurses' Perceptions of Spirituality and Spiritual Care at Five Tertiary Care Hospitals in Riyadh, Saudi Arabia: A Cross-Sectional Study. *Oman Med J, 33*(2), 154-158. doi:10.5001/omj.2018.28

Kb, W. (1998). Clinical scholarship. Professional, ethical, and legal implications for spiritual care in nursing. *Image: Journal of Nursing Scholarship, 30*(1), 81-83.

Kevern, P. (2012). Who can give 'spiritual care'? The management of spiritually sensitive interactions between nurses and patients. *J Nurs Manag, 20*(8), 981-989. doi:10.1111/j.1365-2834.2012.01428.x

Kh, C., & Ds, T. (2008). Spiritual care perspectives of Danish registered nurses. *Journal of Holistic Nursing, 26*(1), 7-14.

Kiaei, M. Z., Salehi, A., Nasrabadi, A. M., Whitehead, D., Azmal, M., Kalhor, R., &

Bahrami, E. S. (2015). Spirituality and spiritual care in Iran: nurses’ perceptions and barriers. *International Nursing Review, 62*, 584–592. doi: 10.1111/inr.12222

Kociszewski, C. (2003). A phenomenological pilot study of the nurses' experience providing spiritual care. *Journal of Holistic Nursing, 21*(2), 131-148.

Kociszewski, C. (2004). Spiritual care: a phenomenologic study of critical care nurses. *Heart & Lung, 33*(6), 401-411.

Kroning, M. (2017). Building a bridge to spiritual care. *Nursing Management, 48*(6), 32-39. doi:10.1097/01.NUMA.0000516488.58802.9d

Ku, Y.-L. (2017). Building Spiritual Care in Nursing: An Overview of Ku's Spiritual Studies in Taiwan. *Journal of Christian nursing : a quarterly publication of Nurses Christian Fellowship, 34*(2), E26-E30. doi:10.1097/CNJ.0000000000000377

Kudubes, A. A., Akıl, Z. K., Bektas, M., & Bektas, İ. (2019). Nurses' Attitudes Towards Death and Their Effects on Spirituality and Spiritual Care. *Journal of religion and health*. doi:10.1007/s10943-019-00927-2

Lee, K., F., Lee, L. Y. K., Lee, J. K. L. (2008). Hong Kong enrolled nurses’ perceptions

of spirituality and spiritual care. *Int Nurs Rev, 55(3)*, 333–340. doi: 10.1111/j.1466-7657.2008.00619.x

Leeuwen, R. van, Cusveller, B. (2004). Nursing competencies for spiritual care. *Journal*

*of Advanced Nursing, 48(3)*, 234–246. doi:10.1111/j.1365-2648.2004.03192.x

Leeuwen, R. van, Tiesinga, L. J., Post, D., Jochemsen, H. (2006). Spiritual care:

implications for nurses’ professional responsibility. *Journal of Clinical Nursing, 15(7)*, 875-884. doi: 10.1111/j.1365-2702.2006.01615.x

Leeuwen, V., Tiesinga, L. J., Middel, B., Post, D., & Jochemsen, H. (2009). The validity

and reliability of an instrument to assess nursing competencies in spiritual care*. Journal of Clinical Nursing, 18(20)*, 2857–2869. doi: 10.1111/j.1365-2702.2008.02594.x

Lundberg, P. C., & Kerdonfag, P. (2010). Spiritual care provided by Thai nurses in

intensive care units. *J Clin Nurs, 19(7-8)*, 1121–1128. doi:10.1111/j.1365-2702.2009.03072.x

Lundmark, M. (2006). Attitudes to spiritual care among nursing staff in a Swedish

oncology clinic. *Journal of Clinical Nursing (Wiley-Blackwell), 15(7)*, 863–874. doi:10.1111/j.1365-2702.2006.01189.x

Martins, A. R., Pinto, S., Caldeira, S., Pimentel, F. L. (2015). Translation and adaptation

of the Spirituality and Spiritual Care Rating Scale in Portuguese palliative care nurses. *Revista de Enfermagem Referência, 4(4)*, 89–97. doi: 10.12707/RIII13129

Marzband, R., Hosseini, S. H., Hamzehgardeshi, Z. (2016). A Concept Analysis of

Spiritual Care Based on Islamic Sources. Religions, 7(61), 1–11. doi:10.3390/rel7060061

Mcbrien, B. (2010). Nurses’ provision of spiritual care in the Emergency Setting – An

Irish Perspective. *Int Emerg Nurs, 18,* 119-126. doi: 10.1016/j.ienj.2009.09.004

Melhem, G. A. B., Zeilani, R. S., Zaqqout, O. A., Aljwad, A. I., Shawagfeh, M. Q., &

Rahim, M. A. A.-. (2016). Nurses’ Perceptions of Spirituality and Spiritual Care Giving: A Comparison Study Among All Health Care Sectors in Jordan. *Indian Journal of Palliative Care, 22(1)*, 42-49. doi:10.4103/0973-1075.173949

Milligan, S. (2011). Addressing the spiritual care needs of people near the end of life.

*Nursing Standard, 26(4)*, 47–56. doi: 10.7748/ns2011.09.26.4.47.c8730

Moosavi, S., Rohani, C., Borhani, F., & Akbari, M. E. (2019). Factors affecting spiritual

care practices of oncology nurses: a qualitative study. *Supportive Care in Cancer, 27(3)*, 901–909. doi:10.1007/s00520-018-4378-8

Moosavi, S., Borhani, F., Akbari, M. E., Sanee, N., & Rohani, C. (2020).

Recommendations for spiritual care in cancer patients: a clinical practice guideline for oncology nurses in Iran. *Support Care in Cancer*, *28*, 5381-5395. doi: 10.1007/s00520-020-05390-4

Musa, A. S., & Pevalin, D. J. (2016). Development of the Arabic Spiritual Care

Intervention-Provision Scale. *Journal of Clinical Nursing, 25(15–16)*, 2275–2284. doi:10.1111/jocn.13174

Musgrave, C. F., & McFarlane, E. A. (2003). Oncology and nononcology nurses' spiritual well-being and attitudes toward spiritual care: a literature review. *Oncol Nurs Forum, 30*(3), 523-527. doi:10.1188/03.ONF.523-527

Musgrave, C. F., & McFarlane, E. A. (2004). Israeli oncology nurses' religiosity, spiritual well-being, and attitudes toward spiritual care: a path analysis. *Oncol Nurs Forum, 31*(2), 321-327. doi:10.1188/04.ONF.321-327

Noormohammadi, M. R., Etemadifar, S., Rabiei, L., Deris, F., Jivad, N., & Masoudi, R.

(2019). Identification of Concepts of Spiritual Care in Iranian Peoples with Multiple Sclerosis: A Qualitative Study*. Journal of Religion & Health, 58(3)*, 949–964. doi:10.0.3.239/s10943-017-0549-z

O´Callaghan, C., Brooker, J., Silva, W. De, Symons, X., & Kissane, D. (2019). Patients’

and Caregivers’ Contested Perspectives on Spiritual Care for Those Affected by Advanced Illnesses: A Qualitative Descriptive Study. *J Pain and Symptom Manage, 58(6),* 977-988, doi:10.1016/j.jpainsymman.2019.08.004

Ozbasaran, F., Ergul, S., Temel, A. B., Gurol Aslan, G., & Coban, A. (2011). Turkish

nurses’ perceptions of spirituality and spiritual care. *Journal of Clinical Nursing, 20(21–22)*, 3102–3110. doi:10.1111/j.1365-2702.2011.03778.x

Pesut, B. (2006). Fundamental or Foundational Obligation? Problematizing the Ethical

Call to Spiritual Care in Nursing. *Advances in Nursing Science, 29(2)*, 125–133. doi: 10.1097/00012272-200604000-00006

Pilaikiat, R., Fongkaew, W., Sethabouppha, H., Phornphibul, P., Voss, J. G. (2016).

Development of a Buddhist Spiritual Care Model for people at the end of life. *Journal of Hospice & Palliative Nursing, 18(4)*, 324-331. doi:10.1097/NJH.0000000000000255

Pipkins, C. M., Rinker, M. A., Curl, E. (2019). Spiritual Care Perceptions of

Baccalaureate Nursing Students. *J Holist Nurs.*, *38(1),* 131-138. doi:10.1177/0898010119887595

Ramenazi, M., Ahmadi, F., Mohammadi, E., Kazemnejad, A. (2014). Spiritual care in

nursing: a concept analysis. International Nursing Review, 1–9. doi: 10.1111/inr.12099

Ross, L. (2006). Spiritual care in nursing: an overview of the research to date. Journal of

Clinical Nursing (Wiley-Blackwell), 15(7), 852–862. doi:10.1111/j.1365-2702.2006.01617.x

Ross, L., Leeuwen, R. Van, Baldacchino, D., Giske, T., Mcsherry, W., Narayanasamy,

A., Downes, C., Jarvis, P., Schep-akkerman, A. (2014). Student nurses perceptions of spirituality and competence in delivering spiritual care: A European pilot study. *Nurse Education Today, 34(5)*, 697-702. doi:10.1016/j.nedt.2013.09.014

Ruder, S. (2013). Spirituality in Nursing: Nurses ’ Perceptions About Providing Spiritual

Care. *Home Healthcare Nurse, 31(7)*, 356–367. doi: 10.1097/NHH.0b013e3182976135

Rushton, L. (2014). What are the barriers to spiritual care in a hospital setting? *British*

*Journal of Nursing 23(7)*, 370–375. doi: 10.12968/bjon.2014.23.7.370

Sanders, B. L., Kopis, S., Moen, C., Pobanz, A., & Volk, F. (2016). Perceptions of

Spirituality and Spiritual Care in Religious Nurses. *Journal of Christian Nursing, 33(4)*, 214–219. doi:10.1097/CNJ.0000000000000308

Sawatzky, R., & Pesut, B. (2005). Attributes of Spiritual Care in Nursing Practice.

*Journal of Holistic Nursing, 23(1)*, 19-33. doi:10.1177/0898010104272010

Silva, O. E. M., Abdala, G. A., Silva, I. A., Meira, M. D. D. (2015). Spiritual care in

nursing practice: nurses’ perception. *J Nurs UFPE on line, 9(8)*, 8817–8824. doi:10.5205/reuol.7696-67533-1-SP-1.0908201508

Sinclair, S., Bouchal, S. R., Chochinov, H., Hagen, N., & McClement, S. (2012). Spiritual

care: how to do it. *BMJ Supportive & Palliative Care, 2(4)*, 319–327. doi:10.1136/bmjspcare-2011-000191

Stephenson, C., Wilson, K. (2004). Does Spiritual Care really help? A Study of patient

perceptions. *Journal of Clinical Nursing, 21(2)*, 26-29. doi: 10.1097/01.CNJ.0000262455.44633.65

Stranahan, S. (2001). Spiritual Perception, Attitudes about Spiritual Care, and Spiritual

Care Practices among Nurse Practitioners. *Western Journal of Nursing Research, 23(1)*, 90-104. doi: 10.1177/01939450122044970

Tiew, L. H., Creedy, D. K., Chan, M. F. (2013). Student nurses’ perspectives of

spirituality and spiritual care. *Nurse Education Today, 33(6)*, 574–579. doi: 10.1016/j.nedt.2012.06.007

Timmins, F., & Caldeira, S. (2017). Understanding spirituality and spiritual care in

nursing. *Nursing Standard, 31(22)*, 50–57. doi: 10.7748/ns.2017.e10311

Tirgari, B., Iranmanesh, S., Cheraghi, M. A., Arefi, A. (2013). Meaning of Spiritual Care:

Iranian Nurses´ Experiences. *Holistic Nursing Practice, 27(4)*, 199–206. doi: 10.1097/HNP.0b013e318294e774

Toker, K., Çınar, F. (2018). Perceptions of Spirituality and Spiritual Care of Health

Professionals Working in a State Hospital. *Religions, 9(10)*, 312. doi: 10.0.13.62/rel9100312

Tüzer, H., Kırca, K., & Özveren, H. (2020). Investigation of Nursing Students’ Attitudes

Towards Death and Their Perceptions of Spirituality and Spiritual Care. *J Relig Health*, *59(4),* 2177-2190. doi: 10.1007/s10943-020-01004-9

Velásquez, S. P. U., Gómez, M. I. L. (2014). “Estar ahí”, significado del cuidado

espiritual: a mirada de los profesionales de enfermeira. *Av Enferm, 32(2)*, 261-270. doi: 10.15446/av.enferm.v32n2.46230

Veloza-Gómez, M., Rodríguez, L. M. de, Guevara-Armenta, C., Mesa-Rodríguez, S.

(2016). The importance of Spiritual Care in Nursing Practice. *Journal of Holistic Nursing, 35(2)*, 1–14. doi: 10.1177/0898010115626777

Vicensi, B. B., Burkhart, L. (2014). Development and Psychometric Testing of New

Instruments to Assess Nurse Practitioners’ Provision of Spiritual Care. Journal of Holistic Nursing, 34(2), 1–11. doi: 10.1177/0898010114544302

Vincensi, B. B., Solberg, M. (n.d.). Assessing the Frequency Nurse Practitioners

incorporate Spiritual Care into Patient-Centered Care. *The Journal for Nurse Practitioners, 13(5)*, 1–8. doi:10.1016/j.nurpra.2017.03.005

Vogel, A., Schep-Akkerman, A. E. (2018). Competence and frequency of provision of

spiritual care by nurses in the Netherlands. *Scand J Caring Sci, 32*, 1314-1321. doi:10.1111/scs.12575

Walker, H., & Waterworth, S. (2017). New Zealand palliative care nurses experiences of

providing spiritual care to patients with life-limiting illness. *International Journal of Palliative Nursing, 23(1)*, 18–26. doi:10.12968/ijpn.2017.23.1.18

Willemse, S., Smeets, W., van Leeuwen, E., Nielen-Rosier, T., Janssen, L., & Foudraine,

N. (2020). Spiritual care in the intensive care unit: An integrative literature research. *Journal of Critical Care, 57*, 55–78. doi: 10.1016/j.jcrc.2020.01.026

Wong, K. F., & Yau, S. Y. (2010). Nurses’ experiences in spirituality and spiritual care

in Hong Kong. *Applied Nursing Research, 23(4)*, 242–244. doi: 10.1016/j.apnr.2008.10.002

Wynne, L. (2013). Spiritual care at the end of life. *Nursing Standard, 28(2)*, 41-45. doi:

10.7748/ns2013.09.28.2.41.e7977
